# Development of acute kidney injury with massive granular casts and microscopic hematuria in patients with COVID-19: two case presentations with literature review

**DOI:** 10.1186/s41100-020-00308-6

**Published:** 2020-12-04

**Authors:** Takuya Fujimaru, Keiki Shimada, Takayuki Hamada, Kimio Watanabe, Yugo Ito, Masahiko Nagahama, Fumika Taki, Shutaro Isokawa, Toru Hifumi, Norio Otani, Masaaki Nakayama

**Affiliations:** 1 Department of Nephrology, St. Luke’s International Hospital, 9-1 Akashi-cho, Chuo-ku, Tokyo, 104-8560 Japan; 2 Department of Emergency and Critical Care Medicine, St. Luke’s International Hospital, 9-1 Akashi-cho, Chuo-ku, Tokyo, 104-8560 Japan

**Keywords:** COVID-19, Acute kidney injury, Vancomycin-induced acute kidney injury, Rhabdomyolysis, Microangiopathy, Urine sediment examination

## Abstract

**Background:**

Complications of acute kidney injury (AKI) are common in patients with coronavirus disease in 2019 (COVID-19). However, clinical characteristics of COVID-19-associated AKI are poorly described. We present two cases of severe COVID-19 patients with AKI.

**Case presentation:**

A 77-year-old woman was suspected of having vancomycin-associated AKI, and a 45-year-old man was suspected of having heme pigment-induced AKI caused by rhabdomyolysis. The granular cast, which is known to be a valuable diagnostic tool for confirming the diagnosis of acute tubular necrosis, was detected in both patients at the onset of AKI. Interestingly, both patients also developed microscopic hematuria at the occurrence of AKI, and one patient had elevated d-dimer and low platelet levels simultaneously.

**Conclusions:**

Some reports suggested that COVID-19-associated microangiopathy contributed to the kidney damage. Therefore, it is possible that our patients might have accompanied renal microangiopathy, and that this pathological background may have caused exaggerated tubular damage by vancomycin or heme pigment. The etiology of AKI in patients with COVID-19 is multifactorial. Superimposition of nephrotoxin(s) and virus-associate intra-renal microangiopathy may be a crucial trigger of kidney injury leading to severe AKI in COVID-19 patients. Therefore, in COVID-19 patients, risk factors for AKI should be taken into consideration to prevent its progression into severe AKI.

## Background

Severe acute respiratory syndrome coronavirus 2 (SARS-CoV-2) was identified to be the cause of a cluster of pneumonia cases in Wuhan, a city in the Hubei Province of China at the end of 2019. It rapidly spread to other provinces in China and around the world. In February 2020, the World Health Organization designated the disease COVID-19, which stands for coronavirus disease in 2019.

Besides severe acute respiratory syndrome, complications of acute kidney injury (AKI) are not uncommon in patients with COVID-19. A recent study of 5449 patients who were hospitalized with COVID-19 in New York revealed that 36.6% of patients developed AKI and 14.3% required renal replacement therapy [1], and to note, AKI is significantly associated with in-hospital mortality in COVID-19 patients [1–4]. Therefore, it is of vital importance to prevent AKI development in this population.

In regard to the pathological mechanisms of AKI development in COVID-19, it is thought that the virus infection is directly linked with kidney injury. This is because angiotensin-converting enzyme 2, a putative receptor for SARS-CoV-2, is expressed in the kidneys of humans [5]. However, clinical characteristics of COVID-19-associated AKI are poorly described.

Herein, two cases are presented of severe COVID-19 patients with AKI. This report suggests the possible role of superimposition of nephrotoxin(s) and virus infection-associated microangiopathy in the kidney for developing severe AKI in COVID-19 patients. This suggests that withholding nephrotoxic agents as much as possible may lessen the risk of AKI occurrence within a clinical setting.

## Case presentation

### Patient 1 (Table 1)

A 77-year-old woman was referred to our hospital suspected of COVID-19 with a 4-day history of dyspnea. Eight days prior to the admission, she had a fever, cough, and sore throat. Her son was diagnosed with COVID-19 2 days before her admission. She had no history of diabetes, hypertension, and/or chronic kidney disease. On the day of admission, she was alert with a body temperature 37.7 °C, blood pressure at 114/66 mmHg, heart rate at 66 per minute, respiration at 24 per minute, and oxygen saturation at 96% (nasal cannula delivering oxygen 4.0 L per minute). The physical examination was unremarkable. The laboratory test revealed an increase in the C-reactive protein (CRP) level. The patient’s renal and liver functions were normal. Using a dipstick urinalysis, 1+ proteinuria and 1+ hematuria were detected. Chest CT without contrast showed ground-glass opacities in bilateral lower lobes. Ceftriaxone and azithromycin were started. Her respiratory condition gradually deteriorated. The SARS-CoV-2 PCR test performed on the day of admission returned positive 2 days later. On the sixth day of admission, she was intubated and received mechanical ventilation, and piperacillin-tazobactam (4.5 g every 6 h) and methylprednisolone (120 mg/day for 3 days) were started. On day 12, due to the possible superimposed infection with fever and increased white blood cell count, blood cultures were taken and vancomycin was administered (loading dose of 2 g and maintenance dose of 1 g twice a day), but it was discontinued on hospital day 15 with negative blood cultures. On hospital day 16, favipiravir was started because of deterioration confirmed by chest CT scan without contrast. On the same day, serum creatinine had started to elevate (trough vancomycin concentration 26.75 ug/mL), despite adequately maintained blood pressure with no clinical signs of hypotension and volume depletion. Urine microscopy detected massive granular casts and mild hematuria with no dysmorphic red blood cells (RBCs) (30–49 RBCs per high-power field [RBCs/HPF]) and a urine dipstick confirmed that there was no presence of proteinuria. The patient was suspected of vancomycin-associated acute tubular necrosis (ATN). Her respiratory condition remained unchanged; however, her serum creatinine level gradually decreased over the course of several days, with no appearance of granular casts and hematuria thereafter. She underwent tracheostomy on hospital day 26, and mechanical ventilation was withdrawn on hospital day 44.

**Table 1:** Physiological and laboratory data of patient 1

Day of admission, days	1	5	8	12	16	19	22	26	34	44
Day of illness, days	8	12	15	19	23	26	29	33	41	51
Renal										
Cr, mg/dL	0.59	0.41	0.62	0.43	2.22	3.30	1.62	0.99	0.56	0.39
Urine volume, ml/day	N/A	890	1775	1815	1320	2160	2515	2325	1230	1020
Serum BUN, mg/dL	17.1	16.9	44.6	34.6	69.2	125.3	79.2	55.5	49.6	37.2
Body weight, kg	55.0 ^c^	54.9	57.6	58.5	56.1	56.4	54.1	55.4	52.0 ^e^	48.4
Urine protein ^a^	1+	N/A	1+	1+	+/− ^d^	+/−	None	+/−	1+	+/−
Urine occult blood ^a^	1+	N/A	None	+/−	+/−	None	+/−	None	+/−	None
Urine RBC, /HPF	N/A	N/A	< 1	5–9	30–49 ^d^	< 1	1–4	1–4	N/A	None
Granular cast ^b^	N/A	N/A	None	None	4+^d^	None	None	1+	N/A	None
Respiratory										
O_2_ Device	NC	MV	MV	MV	MV	MV	MV	MV	MV	VM
PaO_2_/FiO_2_ ratio, mmHg	N/A	123	206	144	148	136	137	137	233	266
Immunology										
WBC, /uL	6800 ^c^	13200	13400	15800	14100	15400	10200	14000	13600	14000
Lymphocyte, /uL	1200 ^c^	790	340	550	280	540	710	490	410	1190
CRP, mg/dL	10.45	20.37	8.02	1.53	16.17	5.92	2.02	16.32	1.51	4.29
Platelet, × 10^3^/uL	133	188	175	158	183	237	195	251	189	262
D-dimer, ug/mL	1.0	6.3	> 100	28.5	24	9.8	12.2	10.4	11.9	3.5

*Cr* Creatine, *BUN* Blood urea nitrogen, *RBC* Red blood cell, *HPF* High-power field, *VCM* Vancomycin, *Conc* Concentration, *PaO*_*2*_ Partial pressure of oxygen, *FiO*_*2*_ Fraction of inspiratory oxygen, *WBC* White blood cell, *CRP* C-reactive protein, *N/A* Not available, *NC* Nasal cannula, *MV* Mechanical ventilation, *VM* Venturi mask

^a^Data by dipstick

^b^Data by microscopy

^c^Data of second day of admission

^d^Data of 17th day of admission

^e^Data of 34th day of admission

### Patient 2 (Table 2)

A 45-year-old man was referred to our hospital with severe respiratory failure and a 10-day history of dyspnea. He had no history of diabetes, hypertension, and/or chronic kidney disease. He was smoking one pack of cigarettes per day. On the day of admission, he was alert with a body temperature of 37.7 °C, blood pressure at 154/110 mmHg, heart rate at 106 per minute, respiration at 36 per minute, and oxygen saturation at 70% with reservoir mask. The laboratory test revealed an increased CRP level. His renal and liver functions were normal. Using a dipstick urinalysis, 1+ proteinuria was detected with no hematuria. Chest CT without contrast showed ground-glass opacities with bilateral peripheral distribution and no pleural effusions. Patient was intubated and received mechanical ventilation, and piperacillin-tazobactam, azithromycin, methylprednisolone, and favipiravir were started empirically due to acute respiratory distress syndrome (ARDS). A SARS-CoV-2 PCR test was performed on hospital day five and returned positive. While the patient’s respiratory condition improved gradually, his high fever still remained with no elevation of white blood cell count or CRP level. On the fifth day of hospitalization, risperidone administration was initiated for delirium. On hospital day eight, a significant increase in serum creatinine kinase accompanying brown colored urine was observed. Using dipstick urinalysis, 2+ proteinuria and 3+ hematuria were detected. In microscopic urinalysis, a few red cells (1–4 RBCs/HPF), myoglobinuria (urine myoglobin concentration was 120,000 ng/mL), and massive granular casts were also detected. Risperidone was discontinued. On hospital day 11, the patient’s serum creatinine was further elevated, although his urine volume was adequately maintained with no significant hypotension and volume depletion. Additionally, he suddenly presented microscopic hematuria with no dysmorphic RBCs (> 50 RBCs/HPF). He was suspected of having heme pigment-associated ATN. His serum creatinine level gradually decreased (peaked at 8.13 mg/dL on hospital day 16), with an eventual disappearance of microscopic hematuria and granular casts. Regarding the pneumonia, the patient discontinued mechanical ventilation on hospital day nine and was discharged on day 33 following two negative PCR tests. After discharge, his renal function recovered to basal level.

**Table 2:** Physiological and laboratory data of patient 2

Day of admission, days	1	5	8	11	15	21	29	40
Day of illness, days	10	14	17	20	24	30	38	49
Renal								
Serum Cr, mg/dL	0.96	1.05	1.82	4.92	8.03	4.35	1.58	0.86
Urine volume, ml/day	1110	1680	1965	1125	2680	2860	3000	N/A
Serum BUN, mg/dL	22.7	43.9	75.9	119	135.8	65.0	15.5	9.8
Body weight, kg	71.7	71.9	71.1	72.8 ^e^	71.9	N/A	66.0	N/A
Urine color	straw	N/A	brown	bloody	bloody	straw	straw	straw
Urine protein ^a^	2+	N/A	2+	3+	2+	+/−	None	None
Urine occult blood ^a^	None	N/A	3+	3+	3+	3+	None	None
Urine RBC, /HPF	N/A	N/A	1–4	> 50	> 50	> 50	None	None
Granular cast ^b^	N/A	N/A	4+	5+	4+	1+	None	None
Urine B2MG, ng/mL	N/A	N/A	4580	N/A	30322 ^f^	7374	608	478
Serum CK, U/L	281	N/A	12901	79508	5547	1220	360	307
Urine myoglobin, ng/mL	N/A	N/A	120000	95000	10000 ^f^	N/A	N/A	N/A
Respiratory								
O_2_ Device	MV	MV	MV	NC	NC	NC	RA	RA
PaO_2_/FiO_2_ ratio, mmHg	211	288	315	382	403	425	N/A	N/A
Immunology								
BT, °C	38.0	41.2	41.9 ^d^	38.2	38.3	37.4	37.3	Afebrile
WBC, /uL	8700 ^c^	7300	7900	19600	8800	11100	3800	2800
Lymphocyte, /uL	1260 ^c^	690	1910	590	440	1000	1220	1850
CRP, mg/dL	15.98	2.95	0.68	0.33	0.79	1.55	0.4	0.49
Platelet, × 10^3^/uL	190	184	100	72	83	220	242	310
d-dimer, ug/mL	16.8	28	> 100	26.8	25.3	N/A	N/A	N/A

*Cr* Creatine, *BUN* Blood urea nitrogen, *RBC* Red blood cell, *HPF* High-power field, *B2MG* Beta-2 microglobulin, *CK* Creatine kinase, *PaO*_*2*_ Partial pressure of oxygen, *FiO*_*2*_ Fraction of inspiratory oxygen, *BT* Body temperature, *WBC* White blood cell, *CRP* C-reactive protein, *N/A* Not available, *MV* Mechanical ventilation, *NC* Nasal cannula

^a^Data by dipstick

^b^Data by microscopy

^c^Data of second day of admission

^d^Data of 7th day of admission

^e^Data of 12th day of admission

^f^Data of 13 h day of admission

## Discussion and conclusions

In this report, patient 1 was suspected of having vancomycin-associated AKI (VA-AKI) from the clinical course. Patient 2 was suspected of having heme pigment-induced AKI caused by rhabdomyolysis. Interestingly, both patients also developed microscopic hematuria at the occurrence of AKI (Table 3). It is not common to find microscopic hematuria in VA-AKI in addition to rhabdomyolysis. Additionally, patient 2 had elevated d-dimer and low platelet levels simultaneously at the same time as serum creatinine started to elevate (Table 2). Although platelet count and d-dimer level could be associated with severity and prognosis of patients with COVID-19 [6], these findings were observed in a COVID-19 patient with thrombotic microangiopathy that was diagnosed by kidney biopsy [7]. Recent studies have suggested that SARS-CoV-2 causes specific manifestations of proximal tubule dysfunction [8]. However, hematuria might not usually be detected in the patients with proximal tubular injury. Therefore, although our patients did not undergo renal biopsy, they also could have accompanied renal microangiopathy.

**Table 3:** Summary of two patients

	Patient 1	Patient 2
Clinical characteristics		
Age, year	77	45
Sex	Female	Male
Hypertension	None	None
Diabetes	None	None
Chronic kidney disease	None	None
Chronic obstructive pulmonary disease	None	None
Days from illness onset to admission, day	8	10
Data of AKI		
Days from illness onset to AKI stage 3 onset, day	23	20
Urine protein at onset of AKI stage 3	+/−	3+
Urine RBC at onset of AKI stage 3, /HPF	30–49	> 50
Urine granular cast	4+	5+
Respiratory status		
Days from illness onset to MV start, day	12	10
Duration of MV, day	40	9

*AKI* Acute kidney injury, *RBC* Red blood cell, *HPF* High-power field, *RRT* Renal replacement therapy, *MV* Mechanical ventilation

A report on autopsy findings from deceased patients with COVID-19 has demonstrated the presence of severe injury of the endothelium in the kidney [9], and it is thought that COVID-19-associated microangiopathy may also contribute to the kidney damage [10]. In a few cohort studies of hospitalized COVID-19 patients, 26.7–48% of them had hematuria as detected by a dipstick [2, 4, 11]. Additionally, a study of 5449 patients who were hospitalized with COVID-19 also highlighted that 40.9% of patients had microscopic hematuria (defined as red blood cells > 5) [1]. Furthermore, in some cohort studies of COVID-19, hematuria was one of the independent risk factors for in-hospital death [2], and elevated d-dimer and low platelet levels were correlated with even worse outcomes [12]. Based on these findings, it is speculated that these patients may have microangiopathy and could be vulnerable to developing severe AKI. In the present two patient cases, it is possible that this pathological background may have caused exaggerated tubular damage by vancomycin or heme pigment.

It is a known fact that critically ill patients are more susceptible to VA-AKI [13]. According to a study on 12,758 medical records in Hong Kong, 1450 patients were identified as VA-AKI, and logistic regression analysis showed that respiratory failure, piperacillin-tazobactam, and meropenem prescription were the risk factors significantly associated with VA-AKI [14]. In the patients with COVID-19, 3–17% developed ARDS [15, 16], and almost all critically ill patients received antibiotics [17, 18]. Therefore, these patients could have a risk of VA-AKI, which suggests the risk of superimposed nephrotoxicity factors in the development of AKI. Additionally, COVID-19 patients may be susceptible to VA-AKI due to the proximal tubular damage caused by SARS-CoV-2.

In patient 2, although the clinical symptoms could be descriptive of neuroleptic malignant syndrome [19], it is still possible that rhabdomyolysis may have been related to SARS-CoV-2 infection. A report of 1099 patients with COVID-19 in China highlighted that two patients had developed rhabdomyolysis [16]. There were five case reports of rhabdomyolysis in COVID-19 patients (Table 4) aged 16–88 years. All patients were male. Among the five patients, three developed AKI [20, 21, 24] and one of three patients received renal replacement therapy [24]. Different pathogeneses of rhabdomyolysis have been suggested; these include direct invasion of the muscle by virus, cytokine storm resulting in muscle damage, and muscle injury by circulating viral toxins [23]. These reports may well suggest the risk of superimposed nephrotoxicity factors in developing AKI as is also the case of antibiotics.

**Table 4:** Case reports of rhabdomyolysis in COVID-19 patients

Age, year	Sex	Onset of rhabdomyolysis, days ^a^	Maximum value of CK, U/L	Maximum value of Cr, mg/dL	Minimum value of Cr, mg/dL	Reference
88	Male	1	13581	1.38	1.09	[20]
49	Male	7	23800	1.18	0.72	[21]
60	Male	15	11842	Normal	Normal	[22]
16	Male	5	457656	0.89	0.68	[23]
37	Male	2	35000	5.0 (with RRT)	2.0	[24]
45	Male	17	79508	8.03	0.86	Patient 2 in this report

*CK* Creatine kinase, *Cr* Creatinine, *RRT* Renal replacement therapy

^a^After onset of COVID-19

In the present two cases, the granular casts were detected in both patients at the onset of AKI (Table 3). A study of 267 AKI patients showed that the granular cast was a valuable diagnostic tool for confirming the diagnosis of ATN [25]. COVID-19 patients are thought to be susceptible to volume depletion and pre-renal AKI due to high fever and/or gastrointestinal losses. In a study of 333 hospitalized patients with COVID-19, 35 patients developed AKI and two of them were diagnosed with pre-renal AKI [11]. The management of ATN includes maintaining an optimal hemodynamic status to ensure renal perfusion. In COVID-19 patients with ARDS, conservative fluid management is recommended [26]. Therefore, urine sediment examination may be a useful tool to make a precise diagnosis and appropriately manage AKI in COVID-19 patients.

The etiology of AKI in patients with COVID-19 is multifactorial. Superimposition of nephrotoxin(s) and virus-associate intra-renal microangiopathy may be a crucial trigger of kidney injury leading to severe AKI in COVID-19 patients. Therefore, in COVID-19 patients, risk factors for AKI should be taken into consideration to prevent its progression into severe AKI.

## Literature review

Nine cohort studies were performed to investigate hematuria in COVID-19 patients (Table 5). Four studies were reported from the same hospital, i.e., Tongji Hospital, Wuhan, China [2, 4, 11, 17, 27]. In nine studies, one study analyzed the patients admitted in the intensive care unit (ICU) [27], and the other studies examined all hospitalized patients [1, 2, 4, 11, 17, 28–30]. The median age ranged from 40 to 60 years. Almost half of the patients were male. In three studies involving the hospitalized patients, 10–30% were in the ICU [1, 2, 30]. The mortality rate ranged from 0 to 70%. Older cohorts tended to have higher mortality rates [1, 2, 17].

**Table 5:** Characteristics of the cohort studies analyzing hematuria in COVID-19 patients

Study	Inclusion period	Institutions	Location	Population	No. of patients	Median age, years	Male (%)	Admitted in ICU (%)	Mortality (%)
Xia et al. [27]	Feb 5–Mar 20, 2020	Tongji Hospital	Wuhan, China	ICU	81	66.6	54 (66.7)	81 (100)	60 (74.1)
Hirsch et al. [1]	Mar 1–Apr 5, 2020	13 hospitals	New York, USA	Hospitalized	5449	64	3317 (60.9)	1395 (25.6)	888 (16.3) ^a^
Hong et al. [28]	Jan 16–Mar 13, 2020	2 hospitals	Sichuan, China	Hospitalized	168	46.7	92 (54.2)	N/A	3 (1.8)
Na et al. [29]	Feb 1–Apr 24, 2020	Chungnam National University Hospital	Daejeon, Korea	Hospitalized	66	45.6	35 (53.0)	N/A	0 (0)
Cheng et al. [2]	Jan 28–Feb 11, 2020	Tongji Hospital	Wuhan, China	Hospitalized	701	63	367 (52.4)	73 (10.4)	113 (16.1)
Chen et al. [17]	Jan 13–Feb 12, 2020	Tongji Hospital	Wuhan, China	Hospitalized	799	68	171 (62)	N/A	113 (14.1) ^b^
Taher et al. [30]	Apr 1–May 31, 2020	Salmaniya Medical Complex	Manama, Bahrain	Hospitalized	73	54	44 (60.3)	23 (31.5)	13 (17.8)
Pei et al. [11]	Jan 28–Feb 9, 2020	Tongji Hospital	Wuhan, China	Hospitalized	333	56.3	182 (54.7)	N/A	29 (8.7)
Li et al. [4]	Jan 6–Feb 21, 2020	Tongji Hospital	Wuhan, China	Hospitalized	193	57	95 (49)	N/A	32 (17) ^c^

*ICU* Intensive care unit, *N/A* Not available

^a^1281 patients were still admitted at publication

^b^525 patients were still admitted at publication

^c^66 patients were still admitted at publication

The prevalence of hematuria and proteinuria is shown in Table 6. One study evaluated only AKI patients [1]. In three studies, urinalysis was performed upon admission [4, 11, 27]. In four studies, the timing of the urinalysis was unknown [2, 17, 28, 30]. In all studies, hematuria was observed in approximately 15–50% patients. Six studies detected hematuria using the urine dipstick test [2, 4, 11, 27, 28, 30], and only two studies used urine sediment examination [1, 29]. Approximately 10–60% patients had proteinuria. Eight studies detected proteinuria using the urine dipstick test [1, 2, 4, 11, 27–30]. In these nine studies, four studies examined the rates of hematuria and proteinuria in AKI patients with COVID-19 (Table 7). The incidence of AKI ranged from 10 to 50%, of which 5–9% required dialysis support [1, 27, 30]. Hematuria was detected in 30–60% of the AKI patients. Approximately 20–80% of AKI patients had proteinuria. All studies used the dipstick to detect proteinuria.

**Table 6:** Hematuria and proteinuria in COVID-19 patients

Study	Hematuria (%)	Definition of hematuria	Proteinuria (%)	Definition of proteinuria	Timing of urinalysis
Xia et al. [27]	10 (27.8) ^a^	> 2+ by dipstick	5 (13.9) ^a^	> 2+ by dipstick	At hospital presentation
Hirsch et al. [1] ^b^	249 (40.9)	RBCs >5/HPF	272 (42.1) ^c^	≥ 2+ by dipstick ^c^	24 h before or 48 h after AKI
Hong et al. [28]	18 (17.5) ^d^	≥ 1+ by dipstick	19 (18.4) ^d^	≥ 1+ by dipstick	N/A
Na et al. [29]	10 (15.2)	RBCs >3/HPF	9 (13.6)	Trace or + by dipstick	During hospitalization
Cheng et al. [2]	118 (26.7) ^e^	≥ 1+ by dipstick	194 (43.9) ^e^	≥ 1+ by dipstick	N/A
Chen et al. [17]	84 (50.6) ^f^	N/A	100 (60.2) ^f^	N/A	N/A
Taher et al. [30]	15 (20.5)	≥ 1+ by dipstick	38 (52.1)	≥ 1+ by dipstick	N/A
Pei et al. [11]	139 (41.7)	≥ Trace by dipstick	219 (65.8)	≥ Trace by dipstick	On the first morning after admission
Li et al. [4]	71 (48.3) ^g^	≥ Trace by dipstick	88 (59.9)	≥ Trace by dipstick	Upon admission

*RBCs* Red blood cells, *HPF* High-power field, *N/A* Not available

^a^Only 36 patients were tested

^b^Only AKI patients were included

^c^Up to 646 of 1993 AKI patients were tested

^d^Only 103 patients were tested

^e^Only 442 patients were tested

^f^Only 166 patients were tested

^g^Only 147 patients were tested

**Table 7:** Hematuria and proteinuria in AKI patients with COVID-19

Study	AKI (%)^a^	Dialysis support (%)	Hematuria^b^ in AKI (%)	Proteinuria^b^ in AKI (%)	Timing of urinalysis
Xia et al. [27]	41 (50.6)	8 (9.9)	6 (31.6) ^c^	3 (15.8) ^b^	At hospital presentation
Hirsch et al. [1]	1993 (36.6)	285 (5.2)	249 (40.9) ^d^	272 (42.1) ^d^	24 h before or 48 h after AKI
Taher et al. [30]	29 (39.7)	7 (9.6)	11 (37.9)	24 (82.8)	N/A
Pei et al. [11]	35 (10.5)	N/A	21 (60.0)	31 (88.6)	On the first morning after admission

*AKI* Acute kidney injury, *RBCs* Red blood cells, *HPF* High-power field, *N/A* Not available, *KDIGO* Kidney Disease: Improving Global Outcomes

^a^AKI definition was based on KDIGO criteria [31]

^b^Definition is shown in Table 6

^c^Only 19 AKI patients were tested

^d^Up to 646 of 1993 AKI patients were tested

The pathophysiology of hematuria and proteinuria in COVID-19 patients remains unclear. Sharma et al. evaluated biopsied kidneys from ten COVID-19 patients with AKI [32]. In their study, all patients had proteinuria and were diagnosed with acute tubular necrosis through kidney biopsy. Interestingly, one patient with massive hematuria (> 50 RBCs/HPF) was also diagnosed with thrombotic microangiopathy. Furthermore, in another study that evaluated biopsy samples of native kidneys from 14 patients with COVID-19, five patients were diagnosed with collapsing glomerulopathy [33]. These studies suggested that COVID-19 affected the kidneys in the tubular, vascular, and glomerular compartments [32].

## Data Availability

All data generated or analyzed during this study are included in this published article.

## Abbreviations

AKI: Acute kidney injury COVID-19 Coronavirus disease in 2019 SARS-CoV-2 Severe acute respiratory syndrome coronavirus 2 CRP C-Reactive protein RBCs/HPF Red blood cells per high-power field ATN Acute tubular necrosis ARDS Acute respiratory distress syndrome VA-AKI Vancomycin-associated AKI
